# Chloroplast genome structure and phylogenetic analysis of *Juniperus chinensis*

**DOI:** 10.1080/23802359.2022.2049015

**Published:** 2022-03-15

**Authors:** Chunyan Chen, Chenhui Song, Youfu Zhang

**Affiliations:** College of Agriculture, Henan University of Science and Technology, Luoyang, PR China

**Keywords:** *Juniperus chinensis*, Cupressaceae, chloroplast genome

## Abstract

The chloroplast genome of *Juniperus chinensis* L. was assembled and annotated in this research. The size of the chloroplast genome is 127,811 bp. It contains a 91,322 bp large single-copy region and a 35,960 bp small single-copy region; it does not contain inverted repeats. The genome encodes 82 protein-encoding genes, 33 tRNAs, and four rRNAs. Phylogenetic analysis showed that *J. chinensis* was closer to congeneric species than those of Cupressaceae. These results provide a genomic basis for further evolutionary research on conifers.

Chinese juniper (*Juniperus chinensis* L. 1767) is an ornamental tree of Cupressaceae family. It mainly grows in the Asian mountains at an elevation of 1400–3512 m a.s.l. (Singh et al. 2018). It is widely used in landscaping, afforestation, and medical purposes because of its antibacterial, antifungal, and antitumor effects (Kim et al. 2016). Its evolutionary relationship with congeneric Cupressaceae species is still uncertain (Miao et al. 2019; Song et al. 2019).

The leaf sample of *J. chinensis* was collected from Luoyang city of Henan Province, China (34°35′58″N, 112°24′53″E). A specimen was identified by Panfeng Dai and kept in the Agriculture College herbarium (Panfeng Dai, dpf@163.com), Henan University of Science and Technology (voucher number YB2021031401). Total DNA was extracted from the sample using E.Z.N.A.^®^ Plant DNA Kit. The whole-genome sequencing was performed on both Illumina NovaSeq 6000 and PacBio Sequel II platforms. Raw data of 5.1 Gb were generated using Illumina, trimmed using Trimmomatic v.0.39 (Bolger et al. 2014), and assembled using GetOrganelle v.1.7.1 (Jin et al. 2020). PacBio data of 74.92 Gb were generated, filtered, and aligned with the assembled Illumina sequence to obtain the target sequence using BWA v.0.7.17 (Li and Durbin 2009). The final full-length sequence was a hybrid assembly of the edited Illumina and Pacbio data using SPAdes v.3.14.1 (Antipov et al. 2016).

The chloroplast genome size of *J. chinensis* is 127,811 bp. The genome has a 91,322 bp large single-copy (LSC) region and a 35,960 bp small single-copy (SSC) region. However, the genome has no inverse repeats (IRs), which is similar to the previously published results on Cupressaceae species (Miao et al. 2019). GC content of the total sequence is 34.97% and the GC contents of LSC and SSC are 34.95% and 35.02%, respectively. The genome encodes 82 protein-coding genes, 33 tRNAs, and four rRNAs.

To elucidate the phylogenetic relationship of *J. chinensis* in Cupressaceae, a maximum-likelihood phylogeny was established from 24 chloroplast genomes using MEGA7 with 1000 bootstrap replicates. (Kumar et al. 2016) (Figure 1). The results indicated that all species of *Juniperus* were clustered into two branches and the species of other genera into another branch. This result confirmed that the genome of *J. chinensis* was similar to congeneric species.

**Figure 1.**
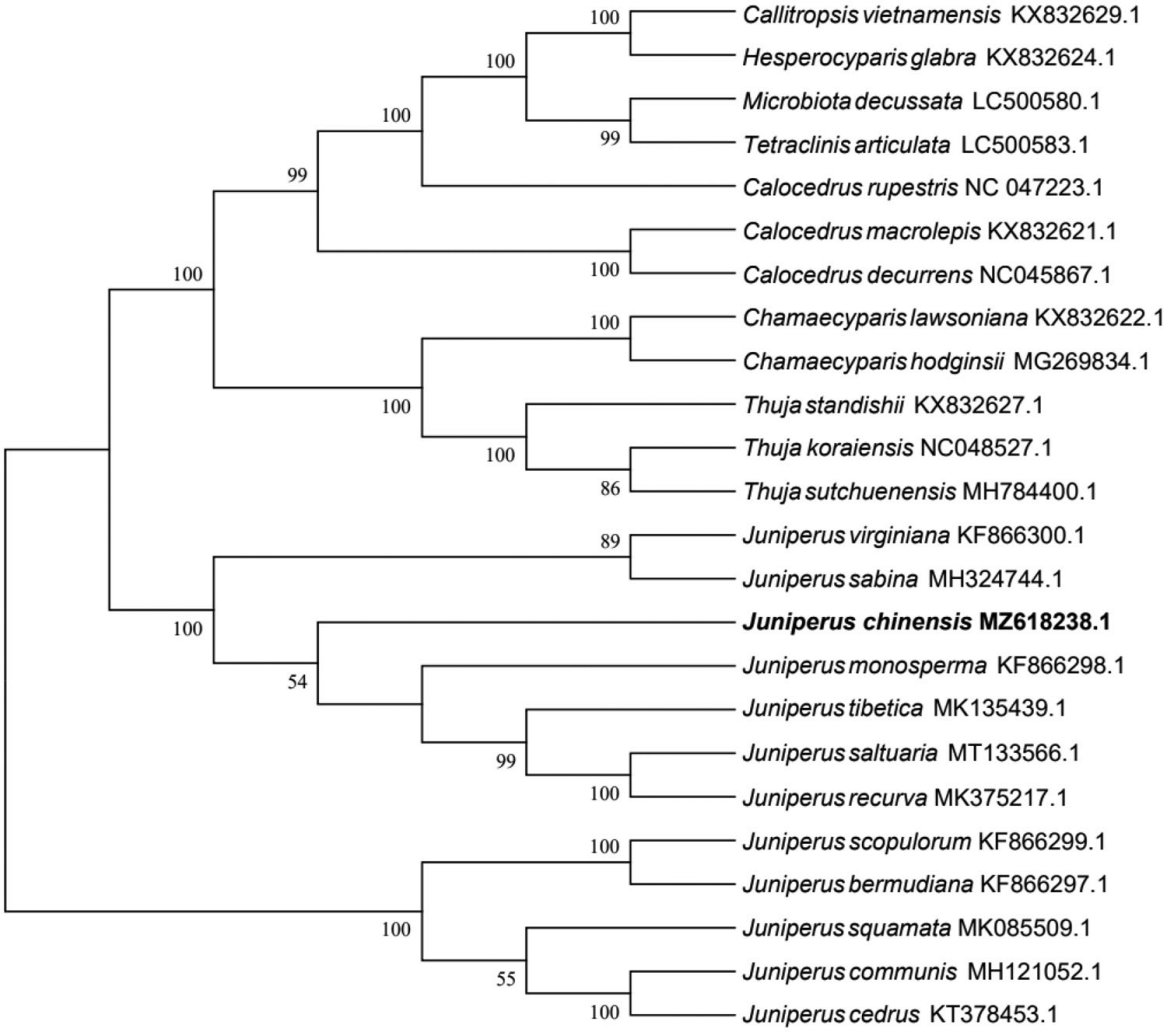
Maximum-likelihood phylogenetic tree established using the chloroplast genome of 24 species.

## Data Availability

The genome sequence data that support the findings of this study are openly available in GenBank of NCBI at https://www.ncbi.nlm.nih.gov/ under the accession no. MZ618238.1. The associated BioProject, SRA, and Bio-Sample numbers are PRJNA777013, SRR16894725 and SRR16894726, and SAMN22830816, respectively.
